# A ***B***ayesian ***R***egularized and ***A***nnotation-***In***formed ***I***ntegrative ***A***nalysis of ***C***ognition (BRAINIAC)

**DOI:** 10.1016/j.dcn.2025.101569

**Published:** 2025-06-25

**Authors:** Rong W. Zablocki, Bohan Xu, Chun-Chieh Fan, Wesley K. Thompson

**Affiliations:** a Herbert Wertheim School of Public Health and Human Longevity Science, University of California San Diego, La Jolla, CA, USA; b Population Neuroscience and Genetics (PoNG) Center, Laureate Institute for Brain Research, Tulsa, OK, USA

**Keywords:** Bayesian modeling, Variance components, Annotations, ABCD Study, Whole-brain analyses

## Abstract

We present the novel Bayesian Regularized and Annotation-Informed Integrative Analysis of Cognition (BRAINIAC) model. BRAINIAC allows for estimation of total variance explained by all features for a given cognitive phenotype, as well as a principled assessment of the impact of annotations on relative enrichment of predictive features compared to others in terms of variance explained, without relying on a potentially unrealistic assumption of sparsity of brain–behavior associations. We validate BRAINIAC in Monte Carlo simulation studies. In real data analyses, we train the BRAINIAC model on resting state functional magnetic resonance imaging (rsMRI) and neuropsychiatric data from the Adolescent Brain Cognitive Development (ABCD) Study and use the trained model in an out-of-study application to harmonized resting-state data from the Human Connectome Project Development (HCP-D), demonstrating a substantial improvement in out-of-study predictive power by incorporating relevant annotations into the BRAINIAC model.

## 1 Introduction

Functional Magnetic Resonance Imaging (fMRI) measures changes in blood oxygenation and blood flow related to neuronal activity, providing researchers with the means to study human brain function *in vivo* (Ombao et al., 2016). fMRI is safe, relatively inexpensive, and has fairly good spatial and temporal resolution. Consequently, over the past two decades fMRI has become an essential and widely-used tool for assessing the neural substrate of human behavior and development.

However, over the past decade or more neuroimaging research has experienced a “replication crisis” (Button et al., 2013), wherein many published associations either do not replicate or have much smaller effect sizes in subsequent studies. It has been hypothesized that this crisis has been caused by a combination of factors, including small effects, inadequate sample sizes, high-dimensional feature spaces (with correspondingly high researcher degrees of freedom) and publication bias towards “significant” associations (Dick et al., 2021). This perfect storm of factors leads to a severe “Winner’s Curse” effect for the field as a whole (van Zwet and Cator, 2021), wherein effect sizes of replication studies tend to be much closer to zero than in initial discovery samples.

In support of this argument, it has been recently shown that effects sizes of brain–behavior relationships estimated from large-sample studies are often considerably reduced compared to those published in the past using data from much smaller samples (Dick et al., 2021, Marek et al., 2022, Miller et al., 2016). Studies such as the Human Connectome Project Development (HCP-D) (Somerville et al., 2018), the Adolescent Brain Cognitive Development (ABCD) Study (Volkow et al., 2018), and the upcoming HEALthy Brain and Child Development (HBCD) Study (Volkow et al., 2021) have greatly increased sample sizes (n≈ 1k to 12k) compared to previous neurodevelopmental cohorts. An important consequence of larger samples is the ability to estimate brain–behavior effect sizes with more power and precision. As shown in van Zwet and Cator (2021), the degree to which subsequent published results replicate is directly related to the power of the discovery sample, determined by the underlying effect sizes and the sample size. Large neurodevelopmental samples such as that available in the ABCD Study are thus crucial for addressing obtaining accurate estimates of effect sizes.

Additionally, analyses leveraging data from these large neuroimaging cohorts suggest that brain–behavior associations, rather than being localized to a sparse set of imaging-derived features (IDFs), are widely distributed across the brain. For example, Zhao et al. (2019) found that whole-brain ridge regression of all vertices outperformed methods (e.g., LASSO) that assumed sparsity for predicting cognitive ability using the N-back task fMRI data in the ABCD Study. Thresholding associations based on levels of significance from small, under-powered studies may be giving a biased and unrealistic view of the degree to which brain–behavior relationships are localized to a small number of IDFs.

This is precisely the scenario that Genome-Wide Association Studies (GWAS) encountered over the last couple of decades (Mathieson, 2021). Candidate gene studies failed to replicate. As sample sizes became larger, it was discovered that the effect size of any single genetic locus was generally very small for most phenotypes, but that effects were widely spread across many loci.

In response, Yang et al. (2011a) proposed a variance-components analysis algorithm, termed Genome-wide Complex Trait Analysis (GCTA) for GWAS data. GCTA is designed to assess the total fraction of variance explained (FVE) for all loci in a GWAS, and can be applied when individual locus effect sizes are tiny and the number of loci far exceeds the number of subjects. GCTA requires subject-level data to fit. (Note, other FVE methods, including LD-Score Regression (Bulik-Sullivan et al., 2015) and GWASH (Schwartzman et al., 2019), work on GWAS summary statistics, typically consisting of publicly-released regression coefficients and p-values for each genetic locus). GCTA is informative in the sense that it captures the total variance explained for a given phenotype by all features *en masse* in a linear additive model. Conceptually, the FVE estimated from GCTA is the expected proportion of variance that could be explained using all (genomic) features simultaneously to predict outcomes in an independent replication sample, if the training sample were infinitely large, i.e., if the regression coefficients were estimated without error.

The original GCTA algorithm does not otherwise inform about which aspects of the features are related to their relative strength of prediction of the given phenotype. GCTA has been modified to allow for partitioning features into disjoint sets (Yang et al., 2011b). This allows researchers to assess relative “enrichment” across subsets of features: while effects may be spread widely across all features, per feature variance explained may be higher on average in some subsets compared to others, perhaps indicating a more central role in producing the phenotype under study. However, these methods do not currently allow for continuous annotations or for multiple characteristics of features to be evaluated simultaneously.

In this paper we describe Bayesian hierarchical variance components model developed for application to fMRI data. The goals of the model are: (1) to estimate the FVE of the complete set of IDFs (which may be of much higher dimension than the number of subjects); and (2) to assess the relative enrichment of variance explained for individual features based on feature-specific “annotations”. Annotations can be multi-dimensional, and of mixed type (i.e., discrete and/or continuous). It is hoped that by expanding the variance components model to allow for feature annotations, that the model will enable expert input and hence become more useful for neurodevelopmental researchers to test hypotheses about which factors are more or less related to explaining brain–behavior associations, while preserving the ability to estimate the FVE of all features without relying on thresholding or unrealistic sparsity assumptions. Additionally, we examine the impact of the *a priori* assumption of independent effects, which may be less tenable in neuroimaging compared to genetic applications.

## 2 Methods

### 2.1 The BRAINIAC model

Here we present the novel Bayesian Regularized and Annotation-Informed Integrative Analysis of Cognition (BRAINIAC) model. BRAINIAC allows for both estimation of total variance explained by all features for a given neurocognitive phenotype, as well as a principled inference of the relative enrichment related to measured characteristics (“annotations”) of some features compared to others, without relying on potentially unrealistic assumptions about the sparsity of brain–behavior associations.

The GCTA algorithm (Yang et al., 2012) is based on a model like the following. Let Y denote a vector of behavioral phenotypes collected from n participants in a Brain-Wide Association Study (BWAS). Let X be the corresponding n×B matrix of IDFs. We assume that Y and the columns of X have been standardized. The GCTA model is given by

Y=Xβ+ϵ

$$\beta ∼\text{N}\left(0,\frac{h^{2}}{B}I\right)$$

(1) $$\epsilon ∼\text{N}\left(0,\left(1-h^{2}\right)I\right)$$

where β is a B-dimensional vector of regression parameters of per-feature association sizes and I is a B×B identity matrix. The FVE of the model is $h^{2}$ (termed the “SNP heritability” in GWAS analyses), where $0\leq h^{2}\leq 1$. Since the total variance of Y is unity by construction, $1-h^{2}$ is the variance left unexplained by the features X. Thus, in the standard GCTA model, each feature explains the same amount of variance on average.

We propose an extension of the GCTA model (1) that allows for a heteroscedastic normal prior for β depending on an M-dimensional vector Z for each feature. The generative model is the same as Eq. (1), but with the prior on β instead given by:

(2) $$\beta ∼\text{N}\left(0,h^{2}\Psi \right)$$

$$\Psi =\frac{\text{diag}\left\{\exp\left(Z_{1}^{T}\alpha \right),\exp\left(Z_{2}^{T}\alpha \right),\ldots ,\exp\left(Z_{B}^{T}\alpha \right)\right\}}{\overset{B}{\underset{k=1}{\sum }}\exp\left(Z_{k}^{T}\alpha \right)}.$$

The variance of β is thus comprised of two components: as before, $h^{2}$ quantifies the FVE explained by all features, whereas the B×B diagonal matrix Ψ quantifies how annotations Z modulate the variance explained by individual features. Here, $Z={\left(Z_{1},Z_{2},\ldots ,Z_{B}\right)}^{T}$ is a B×M matrix, where M is the number of the annotations per feature and α is an M-dimensional vector of annotation weights. We assume the weights are unknown and hence need to be learned from the data. The matrix Ψ is scaled (so that the diagonal elements lie between 0 and 1 and sum to unity) to ensure the sum of the variances of β across all features is $h^{2}$. If α=0, the prior of β reduces to $\text{N}\left(0,\frac{h^{2}}{B}I\right)$, which is equivalent to the GCTA model. Alternatively, when α≠0, the feature variances are heteroscedastic, so that some features account for more variance than others depending on the levels of $Z_{k}$, for 1≤k≤B.To complete the Bayesian model, we assign a normal prior to α

(3) $$\alpha ∼\text{N}\left(0,\sigma_{\alpha }^{2}I\right).$$

The FVE $h^{2}$ is given a Beta prior defined on the interval [0,1],

(4) $$h^{2}∼\text{Beta}\left(a,d\right)$$

where $\sigma_{\alpha }^{2}$, a and d are hyperparameters. In applications, we often choose a large value of $\sigma_{\alpha }^{2}$ for a diffuse prior on α. If a and d are both set to unity, the prior is a Uniform distribution on interval [0,1]. If there were prior information about the amount of variance explained by all brain features, we could set a and d appropriately to obtain a specified prior mean and variance. Bayesian inference is performed via a Markov Chain Monte Carlo (MCMC) sampling algorithm, implemented in Python and described in the Supplementary Materials. The Python code is publicly available on GitHub at https://github.com/nidaye1999/BRAINIAC.

## 3 Results

The Adolescent Brain Cognitive Development${}^{\text{SM}}$ (ABCD) Study is the largest single-cohort long-term longitudinal study of neurodevelopment and child and adolescent health in the United States. The ABCD Study® collects observational data to characterize US population trait distributions and to assess how biological, psychological, and environmental factors (including interpersonal, institutional, cultural, and physical environments) can relate to how individuals live and develop in today’s society. The study has primary data collection at 21 research institutes across the USA. Participants include n=11,880 youths aged 9–10 at baseline. Details of the study design are given in Garavan et al., 2018, Dick et al., 2021. The neuroimaging protocol, including the resting state MRI (rsMRI), has been described elsewhere (Casey et al., 2018). We employed the ABCD Study data as the basis for Monte Carlo simulations as well as real data analyses, as detailed below. Data were obtained from the ABCD Study Curated Data Release 4.0 (https://nda.nih.gov/DOI 10.15154/1,523,041).

### 3.1 Monte Carlo simulations

#### 3.1.1 Performance evaluation

We implemented Monte Carlo simulation studies to evaluate the BRAINIAC model performance. The goal of the Monte Carlo simulations was to examine if the posterior distributions of parameters α, $h^{2}$ and β were centered on their true values, and that their 95% posterior credible intervals (CI’s) contained the true parameter close to their nominal coverage level (i.e., 95% of the time). To make these simulations as realistic as possible, we extracted the rsMRI data at baseline, when participants were 9–10 years of age. If these data were missing at baseline for a given participant, we used their data from their 2-year follow-up visit if those were available.

The rsMRI features of interest were the Fisher z-transformed Pearson correlations (“connectivities”) of activation time courses between pairs of cortical regions defined by the Gordon parcellation (Gordon et al., 2016). This resulted in an approximately 1:5 ratio of participants to features. We annotated these connectivities based on whether edges connected two nodes within a specific network, where networks were chosen from a subset of ten of the networks presented in Gordon et al. (2017). Next, we reduced the set of features, only retaining those features that fell onto one of the ten networks, resulting in B= 14,019. To preserve the 1:5 ratio of participants to features, we subsequently randomly sampled n=2,804 participants, yielding the final n×B matrix X used for the simulation studies.

The prior distribution β from Eqs. (1), (2) assume a diagonal covariance matrix, i.e., that the β are *a priori* independent. (Note, however, the conditional posterior of β, given by Supplementary Equation (2), is normal but with a non-diagonal covariance matrix). In order to assess the sensitivity of posterior inferences to this choice of prior, simulations were performed with β drawn from first-order autoregressive covariance matrices. Specifically, let k and $k^{\prime }$ denote indices of two IDFs. In our Monte Carlo simulations, the covariance of $\beta_{k}$ and $\beta_{k\prime }$ was then given by

$$\text{Cov}\left(\beta_{k},\beta_{k\prime }\right)=h^{2}\sqrt{\Psi_{k,k}\times \Psi_{k^{\prime },k^{\prime }}}\times \rho^{\left|k-k^{\prime }\right|},$$

where $h^{2}\Psi_{k,k}$ (resp. $h^{2}\Psi_{k^{\prime },k^{\prime }}$) is the prior variance of $\beta_{k}$ (resp. $\beta_{k^{\prime }}$) from Eq. (2) and 0≤ρ<1. Choosing ρ=0 corresponds to the BRAINIAC model. We varied ρ to equal 0,0.2,0.4,0.6 and 0.8. In addition, we set $h^{2}$ to values 0.2,0.4,0.6 and 0.8. For each of the 5×4=20 combinations of ρ and $h^{2}$, we set two of the ten-dimensional annotation weights $\alpha_{1}=-1.13$ and $\alpha_{2}=0.38$ to represent enriched and unenriched effects. Finally, Y was generated from Eq. (2) described in Section 2.1. The MCMC algorithm was implemented with 10 chains per setting.

**Fig. 1:**
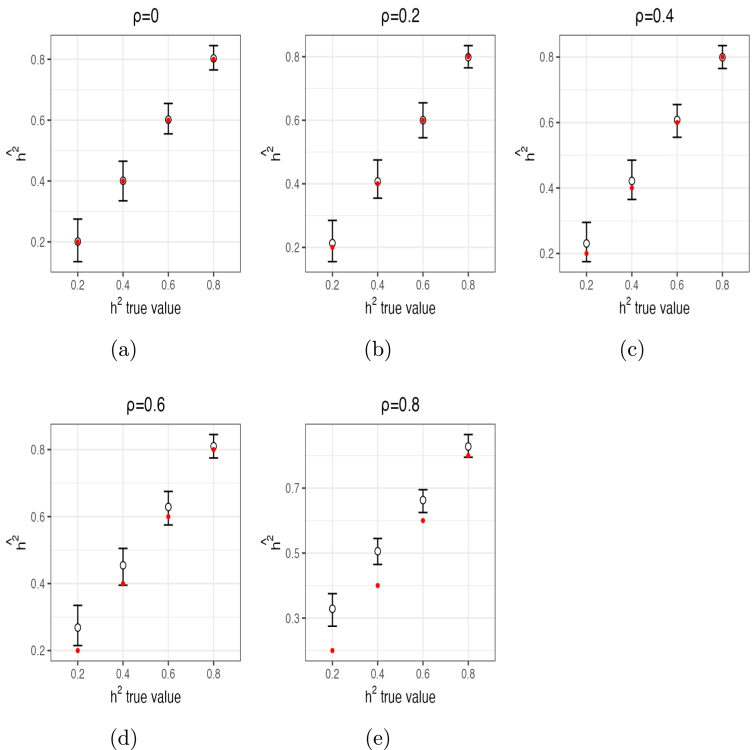
Mean estimates (circles) of $h^{2}$ and 95% posterior CIs (error bars) from 10 chains per setting (red dots indicate true values of $h^{2}$).

**Fig. 2:**
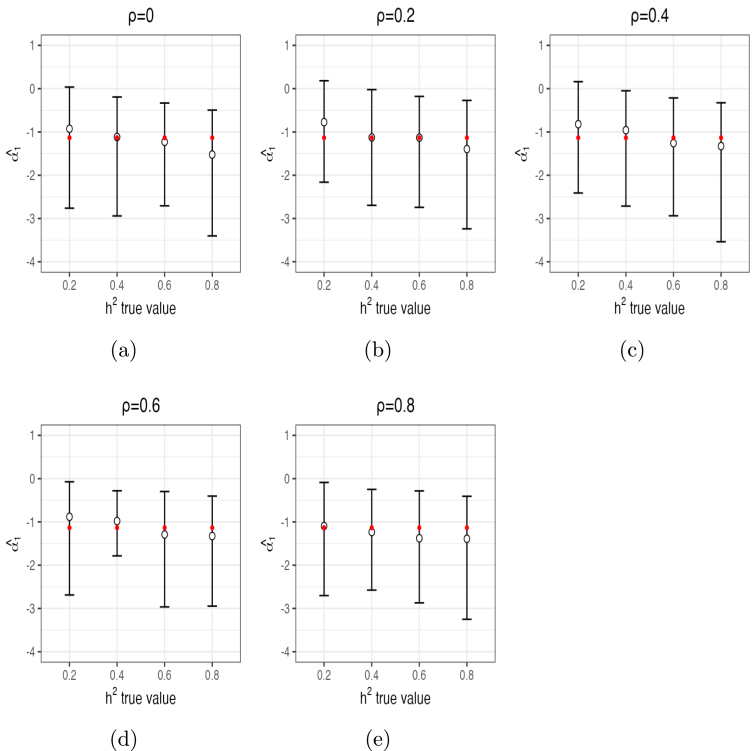
Mean estimates (circles) of $\alpha_{1}$ and 95% posterior CIs (error bars) from 10 chains per setting (red dots indicate true values of $\alpha_{1}$).

**Fig. 3:**
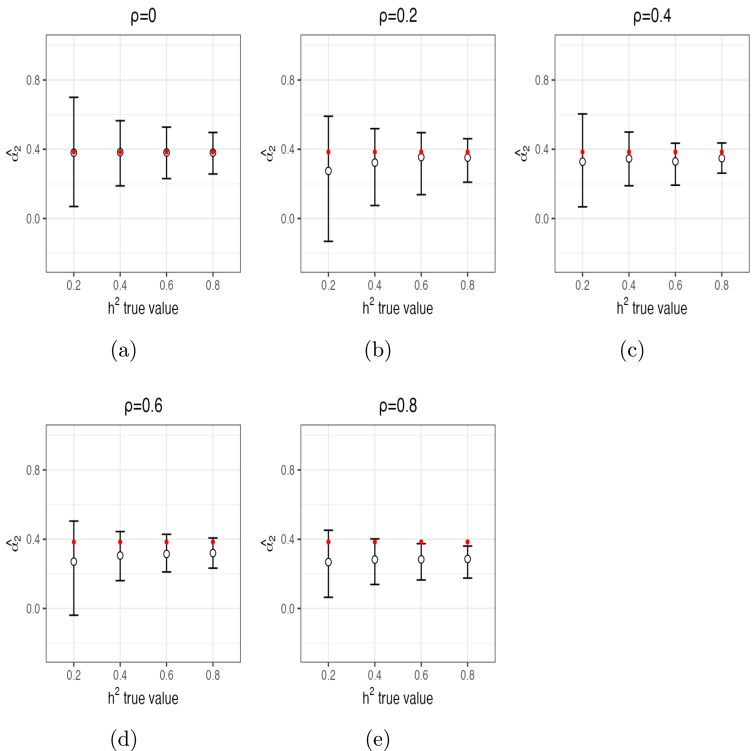
Mean estimates (circles) of $\alpha_{2}$ and 95% posterior CIs (error bars) from intermix of 10 chains per setting (red dots indicate true values of $\alpha_{2}$).

**Fig. 4:**
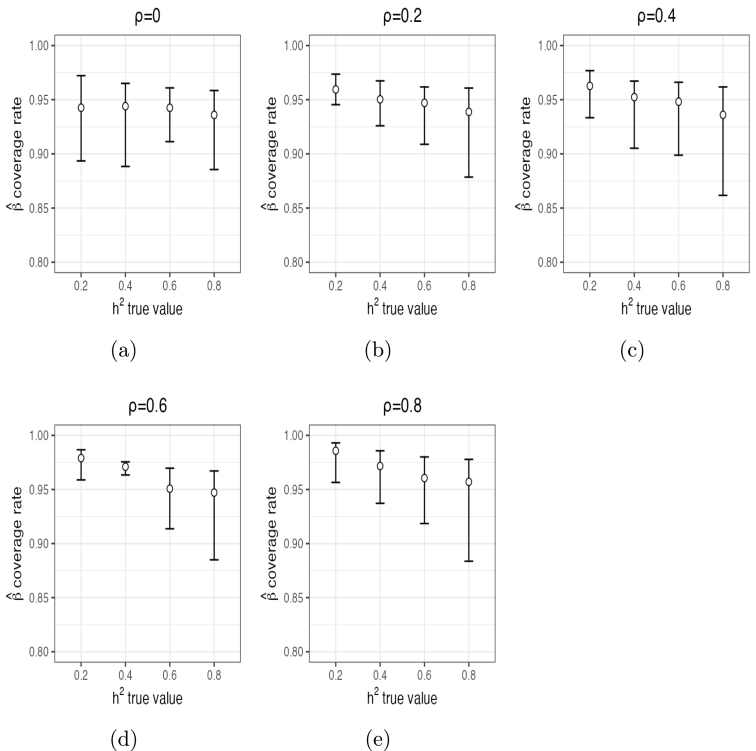
Means (circles) and their 95% posterior CIs (error bars) of $\overset{ˆ}{\beta }$ coverage rates for the true values based on 10 chains per setting.

For $h^{2}$, $\alpha_{1}$ and $\alpha_{2}$, posterior mean estimates and 95% posterior CIs from 10 independent chains are shown in Figs. 1, 2 and 3, respectively. These figures demonstrate that the estimates of these parameters captured their true values accurately in most of the settings, especially with ρ≤0.4 and/or $h^{2}\geq 0.6$. For ρ≤0.4, posterior means were quite accurate and the 95% CI’s all contained the true values of all parameters. For ρ=0.6, estimates of $h^{2}$ were biased upwards for smaller values (average posterior mean estimate of 0.27 when $h^{2}=0.2$ and average posterior mean estimate of 0.45 when $h^{2}=0.4$). For ρ=0.8, estimates of $h^{2}$ were also biased (e.g., average posterior mean estimate of 0.33 when $h^{2}=0.2$ and average posterior mean estimate of 0.51 when $h^{2}=0.4$). Thus, small to moderate departures from independence of β only had modest impacts on the estimates of $h^{2}$. Moderately large departures (ρ=0.6) led to small upward biases for small values of $h^{2}$, and large departures (ρ=0.8) led to more substantial upward biases, especially for small values of $h^{2}$. In contrast, estimates of $\alpha_{1}$ (Fig. 2) were accurate even for large values of ρ. The same is true for $\alpha_{2}$ (Fig. 3), although this parameter was slightly upwardly biased for ρ=0.6 and a bit more for ρ=0.8. The number of times out of the ten chains that the true value for each of the three parameters for each setting was contained in the 95% CI’s is given in Supplementary Section (1.3) Table (1). Means and 95% posterior CIs of the coverage rates for β are shown in Fig. 4. Overall, the average coverage rates of β ranged from 94% to 98% across all settings, matching nominal rates well.

**Fig. 5:**
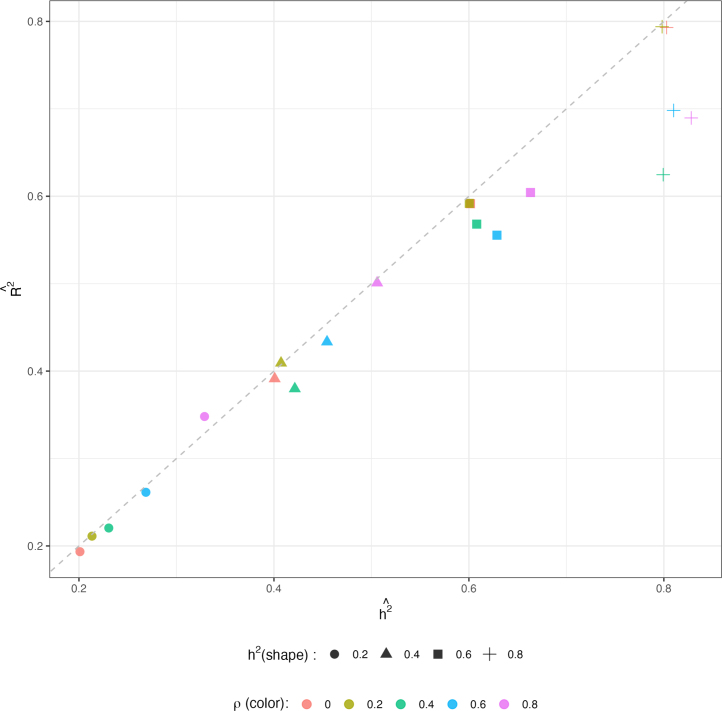
Mean $\overset{ˆ}{h^{2}}$ and $\overset{ˆ}{R^{2}}$ from intermix 10 chains per setting (combination of the color and shape indicates a setting of ρ and $h^{2}$ true values; for example, green square implies ρ=0.4 and $h^{2}=0.6$; hot pink plus sign implies ρ=0.8 and $h^{2}=0.8$). Dash line is the diagonal line.

We also computed ${\overset{ˆ}{R}}^{2}=\text{var}\left(X\overset{ˆ}{\beta }\right)/\text{var}\left(Y\right)$, the empirical variance explained by all features directly from the posterior regression. This should be approximately equal to the theoretical variance explained, i.e., $h^{2}$. The alignment between mean values of $\overset{ˆ}{h^{2}}$ and $\overset{ˆ}{R^{2}}$ from inter-mixed ten chains per setting are shown in Fig. 5. The values match well (lie on the diagonal) until both $h^{2}=0.8$ and ρ≥0.4, indicating that within-sample prediction accuracy of the BRAINIAC model closely tracks the true $h^{2}$ even under moderately large departures from independence, though modestly underestimating $h^{2}$ when both $h^{2}$ and ρ are large.

#### 3.1.2 Out-of-sample prediction performance

We next evaluated the drop-off in performance in an out-of-sample prediction for a given $h^{2}$ estimate. For this simulation, we used the simplified prior of β from Eq. (1). The true value of $h^{2}$ was set to 0.32, similar to the real-data analyses of crystallized intelligence described below. The matrix of rsMRI X connectivities was again selected from the ABCD Study baseline data and was randomly split into a training set ($X_{tr}$) and test set ($X_{ts}$) ten different times. Each $X_{tr}$ had a dimension of 8,665 × 46,360 and each $X_{ts}$ had a dimension of 320 × 46,360. The outcome variables, $Y_{tr}$ and $Y_{ts}$, were generated from Eq. (1) based on standardized $X_{tr}$ and $X_{ts}$, respectively. The BRAINIAC algorithm was applied to the 10 training sets to obtain parameter estimates ${\overset{ˆ}{\beta }}_{tr}$ and ${\overset{ˆ}{h^{2}}}_{tr}$, which were the median values of the MCMC chains. In-sample estimate ${\overset{ˆ}{h^{2}}}_{tr}$ ranged from 0.30 to 0.36, indicating reliable and stable estimates of $h^{2}$. The squared correlation between $X_{ts}{\overset{ˆ}{\beta }}_{tr}$ and $Y_{ts}$ represented the out-sample $h^{2}$ estimate from the test sets, which ranged from 0.10 to 0.21 with median value of 0.15. This result indicates the expected drop-off in out-of-sample prediction performance due to noisy estimates of β from finite training and test samples.

### 3.2 Application to ABCD study data

#### 3.2.1 rsMRI predicting crystallized intelligence

For the real data analyses, we again used the resting-state MRI Fisher z-transformed Pearson correlations of activation time courses between pairs of Gordon parcels (Gordon et al., 2016). The first neurocognitive phenotype Y we used was the crystallized intelligence composite score (age uncorrected) assessed using the NIH Toolbox (Akshoomoff et al., 2013). We used rsMRI and NIH Toolbox data collected at the baseline visit. We retained participants passing quality control metrics and without any missing values in resting state features (X) and crystallized intelligence (Y). We residualized both X and Y on age at baseline, sex at birth, the first ten principal components of genetic ancestry and MRI scanner instance. The residualized X and Y were then standardized to have zero (column) means and unit variances. The final sample size was n=5,643 (male: 2,737, mean [min, max] age in months: 120.0 [107,132]; female: 2,906, age: 119.5 [107–132]). The number of features was B=61,776, yielding an approximate 11:1 ratio of features B to participants n. The annotations Z we used in these analyses were the assignment of cortical vertices into within-network labels, using the 13 resting state networks described in Gordon et al. (2017) including: Auditory, Cingulo-Opercular, Cingulo-Parietal, Default Mode, Dorsal Attention, Fronto-Parietal, Retrosplenial-Temporal, Somato-motor Mouth, Somato-motor Hand, Salience, Subcortical, Ventral Attention, and Visual. There was also an annotation into a “None–None” category, consisting of edges between nodes not contained in any of these thirteen named networks. The percentages of edges in each of within-network annotations are displayed in Supplementary Section (1.4) Table (2). Note, the majority of edges do not fall within any of these networks, and hence most annotations $z_{k}=0$.

We first applied these annotations to the BRAINIAC model one at a time; in other words, for each run M=1 (single-annotation model). For each model, we ran six MCMC chains per annotation; each chain was run for 10,000 iterations with a 3,000 burn-in period and a thinning rate of ten. Posterior parameter estimates of single-annotation model from within-network are presented in Table 1. Estimates shown are the overall median value and 95% posterior credible intervals (CIs). Estimates of $h^{2}=0.37$ was highly consistent across all models. This indicates that the set of all resting state features in the model in total explains 37% of the variance of crystallized intelligence in ABCD participants after residualizing for the covariates listed above. This is a much higher total effect size than has been reported by individual features in the ABCD Study and other large neuroimaging studies (Marek et al., 2022). Note, although ${\overset{ˆ}{R}}^{2}=\text{var}\left(X\overset{ˆ}{\beta }\right)/\text{var}\left(Y\right)$ is not a parameter in the BRAINIAC model, it is the empirical variance explained by the features from the regression and should be close to $h^{2}$, which in fact it is here (see Table 1). Only one within-network annotation (Default Mode Network, DMN) was significantly enriched for associations, with α=0.19 (95% CI: 0.07,0.27). Thus, each edge in the DMN on average explains exp(0.19)=1.21 times as much variance compared to edges outside of the DMN. Despite being enriched for associations, most of the resting state signal for crystallized intelligence lies outside of the DMN.

Next, we examined the predictive power of the BRAINIAC model in an out-of-study application to data taken from the Human Connectome Project Development (HCP-D) study. We extracted the coefficients from the BRAINIAC model applied to the ABCD Study data (1) with no annotations (β); and (2) using DMN as an annotation ($\beta_{DMN}$). These coefficients were then applied to the resting state connectivities $X_{HCP}$ of n=320 HCP-D participants (male: 157, age: (186.2 [97–262]; female: 163, age: 183.0 [97–263]) processed similarly to the ABCD Study training sample, to produce *polyvertex scores*: $\mathrm{PVS}=X_{HCP}\beta$ and ${\mathrm{PVS}}_{DMN}=X_{HCP}\beta_{DMN}$. These were then placed within separate linear regression models, including age and sex as covariates, to predict their crystallized intelligence scores. The PVS explained 11% of the variance in crystallized intelligence over and above sex and age, whereas ${\mathrm{PVS}}_{DMN}$ explained 17% of the variance in crystallized intelligence. Thus, inclusion of the DMN annotation in the BRAINIAC model improves its out-of-study predictive performance by nearly 50% over the model with no annotations.

**Table 1:** Estimates^a^ from single-annotation model with 14 within-network annotations (phenotype : Crystallized Intelligence).

Annotations	$\overset{ˆ}{\alpha }$	${\overset{ˆ}{h}}^{2}$	${\overset{ˆ}{R}}^{2}$
Auditory–Auditory	−0.03 (−0.39,0.10)	0.37 (0.34,0.41)	0.37 (0.34,0.41)
CinguloOperc–CinguloOperc	0.03 (−0.38,0.18)	0.37 (0.34,0.41)	0.37 (0.34,0.41)
CinguloParietal–CinguloParietal	0.00 (−0.67,0.07)	0.37 (0.34,0.41)	0.37 (0.34,0.41)
Default–Default	0.19 (0.07,0.27)	0.37 (0.33,0.41)	0.37 (0.34,0.40)
DorsalAttn–DorsalAttn	0.02 (−0.29,0.18)	0.37 (0.34,0.41)	0.37 (0.34,0.41)
FrontoParietal–FrontoParietal	0.00 (−0.41,0.14)	0.37 (0.34,0.41)	0.37 (0.34,0.41)
None–None	−0.23 (−0.75,0.05)	0.37 (0.34,0.41)	0.37 (0.34,0.41)
RetrosplenialTemporal–RetrosplenialTemporal	−0.23 (−0.83,0.04)	0.37 (0.34,0.41)	0.37 (0.34,0.41)
SMhand–SMhand	0.04 (−0.36,0.17)	0.37 (0.34,0.41)	0.37 (0.34,0.41)
SMmouth–SMmouth	−0.23 (−0.75,0.00)	0.37 (0.34,0.41)	0.37 (0.34,0.41)
Salience–Salience	−0.31 (−0.88,0.01)	0.37 (0.34,0.41)	0.37 (0.34,0.41)
Subcort–Subcort	−0.18 (−0.94,0.09)	0.37 (0.34,0.41)	0.37 (0.34,0.41)
VentralAttn–VentralAttn	0.05 (−0.62,0.17)	0.37 (0.34,0.41)	0.37 (0.34,0.41)
Visual–Visual	0.03 (−0.25,0.18)	0.37 (0.34,0.41)	0.37 (0.34,0.41)

a Estimates ($\overset{ˆ}{\alpha }$, ${\overset{ˆ}{h}}^{2}$, ${\overset{ˆ}{R}}^{2}$) shown are the overall median value and 95% posterior credible interval (CI) from the intermix of 6 chains per annotation. ${\overset{ˆ}{R}}^{2}=\text{var}\left(X\overset{ˆ}{\beta }\right)/\text{var}\left(Y\right)$ is calculated for each chain at each collected iteration for all 6 chains per annotation, the overall median and 95% CI per annotation are reported. $\overset{ˆ}{\beta }$ is a vector estimate of β.

Note, the out-of-study performance of PVS and ${\mathrm{PVS}}_{DMN}$ (11% variance explained without annotation, 17% variance explained with DMN as an annotation) is lower than $h^{2}=0.37$. This is to be expected because of the finite sample size of the ABCD Study training sample. The parameter $h^{2}$ represents the theoretical upper bound of the out-of-sample variance explained if the training sample size were infinite (and hence β was estimated with no error). This drop-off in performance is similar to the performance drop-off for the training and test sets observed in the Monte Carlo simulations presented above.

#### 3.2.2 rsMRI predicting the P-factor

We next performed the same BRAINIAC analyses, but replacing crystallized intelligence with a measure of psychopathology based on the Child Behavior Checklist (CBCL) (Achenbach and Edelbrock, 1991). The CBCL has eight syndrome scales assessing different aspects of psychopathology (Anxious/Depressed, Depressed, Somatic Complaints, Social Problems, Thought Problems, Attention Problems, Rule-Breaking Behavior, Aggressive Behavior). We placed these in a Bi-Factor Structural Equation Model to obtain a unidimensional “P-factor” measure, using the lavaan package in R (Rosseel et al., 2017). Higher scores on the P-factor indicate higher levels of psychopathology. Code for computing the P-factor from the ABCD Study Release 4.0 data is available on GitHub (https://github.com/nidaye1999/BRAINIAC).

We used the exact analysis pipeline as described above for crystallized intelligence. The final sample size was n=5,721 (slightly differing from the crystallized intelligence analyses due to differential missingness). The BRAINIAC results are presents in Table 2. The estimates of $h^{2}=0.09$ (CI = [0.07,0.12]) were highly stable across the different BRAINIAC models. Thus, rsMRI features were less predictive of psychopathology than crystallized intelligence. No within-network annotations reached nominal significance in this application.

We performed the same out-of-study replication analysis using the harmonized HCP-D rsMRI data as described above but replacing crystallized intelligence with the P-factor computed from the CBCL syndrome scales assessed on the HCP-D participants. Since none of the annotations reached significance, we computed only the PVS score without annotations. This resulted in FVE = 1.2%, considerably lower than the predictive performance of PVS and ${\text{PVS}}_{DMN}$ for crystallized intelligence, as expected corresponding to the lower $h^{2}$ estimate for the P-factor.

**Table 2:** Estimates^a^ from single-annotation model with 14 within-network annotations (phenotype : Child Behavior Checklist P-factor).

Annotations	$\overset{ˆ}{\alpha }$	${\overset{ˆ}{h}}^{2}$	${\overset{ˆ}{R}}^{2}$
Auditory–Auditory	−0.08 (−0.46,0.17)	0.09 (0.07,0.12)	0.09 (0.07,0.12)
CinguloOperc–CinguloOperc	−0.01 (−0.41,0.22)	0.09 (0.07,0.12)	0.09 (0.07,0.12)
CinguloParietal–CinguloParietal	−0.28 (−1.04,0.00)	0.09 (0.07,0.12)	0.09 (0.07,0.12)
Default–Default	0.18 (−0.27,0.32)	0.09 (0.07,0.12)	0.09 (0.07,0.12)
DorsalAttn–DorsalAttn	−0.03 (−0.53,0.21)	0.09 (0.07,0.12)	0.09 (0.07,0.12)
FrontoParietal–FrontoParietal	0.18 (−0.14,0.24)	0.09 (0.07,0.12)	0.09 (0.07,0.12)
None–None	−0.09 (−0.52,0.21)	0.09 (0.07,0.12)	0.09 (0.07,0.12)
RetrosplenialTemporal–RetrosplenialTemporal	−0.13 (−0.70,0.09)	0.09 (0.07,0.12)	0.09 (0.07,0.12)
SMhand–SMhand	0.04 (−0.52,0.26)	0.09 (0.07,0.12)	0.09 (0.07,0.12)
SMmouth–SMmouth	−0.14 (−0.65,0.09)	0.09 (0.07,0.12)	0.09 (0.07,0.12)
Salience–Salience	−0.29 (−0.94,0.04)	0.09 (0.07,0.12)	0.09 (0.07,0.12)
Subcort–Subcort	−0.01 (−0.82,0.18)	0.09 (0.07,0.12)	0.09 (0.07,0.12)
VentralAttn-VentralAttn	−0.16 (−0.77,0.13)	0.09 (0.07,0.12)	0.09 (0.07,0.12)
Visual–Visual	−0.08 (−0.52,0.21)	0.09 (0.07,0.12)	0.09 (0.07,0.12)

a Estimates ($\overset{ˆ}{\alpha }$, ${\overset{ˆ}{h}}^{2}$, ${\overset{ˆ}{R}}^{2}$) shown are the overall median value and 95% posterior credible interval (CI) from the intermix of 6 chains per annotation. ${\overset{ˆ}{R}}^{2}=\text{var}\left(X\overset{ˆ}{\beta }\right)/\text{var}\left(Y\right)$ is calculated for each chain at each collected iteration for all 6 chains per annotation, the overall median and 95% CI per annotation are reported. $\overset{ˆ}{\beta }$ is a vector estimate of β.

## 4 Discussion

Here, we have presented the novel BRAINIAC model for analyzing whole-brain data associations with neurocognitive phenotypes. This model allows for estimation of the overall FVE by all imaging features simultaneously, while also allowing for systematic principled inferences regarding differences in FVE as captured by feature-level annotations. Thus, the BRAINIAC model goes beyond variance components models such as GCTA, that simply report the FVE explained by all features. By incorporating as annotations-specific aspects of the imaging derived features, we can partially localize effects and rigorously estimate and test for relative enrichment of some features compared to others.

In an application of the BRAINIAC model to the ABCD Study baseline resting state MRI data, we found that the edge connectivities in total accounted for 37% of the variance of crystallized intelligence, a much higher effect size than has been reported using IDFs individually with the ABCD Study data (Marek et al., 2022). One of fourteen within-network annotations (namely the Default Mode Network) enriched for associations with crystallized intelligence. However, since the within-DMN edges only account for 1.33% of all features in the model, the DMN still accounts for only a small fraction of the total variance accounted for by all features, underscoring the wide-spread nature of many brain–behavior associations. Nevertheless, including the DMN as an annotation lead to an over 50% improvement in out-of-study prediction accuracy compared to prediction not using this as an annotation, highlighting the potential of BRAINIAC for leveraging data from large studies such as the ABCD Study for training algorithms for powering predictions in smaller studies.

Applying BRAINIAC to the P-factor, a measure of psychopathology, resulted in an estimated $h^{2}=0.09$, considerably lower than for crystallized intelligence. This conforms with results from other studies examining the relationship between psychopathology and brain function using the ABCD Study rsMRI data (Karcher et al., 2021). None of the within-network annotations reached nominal significance in this example.

Simulations confirmed the good performance of BRAINIAC even when the number of features is much larger than the number of participants. A key aspect of the BRAINIAC model is the prior specification on β, which is normal with a diagonal covariance matrix. In simulations we assessed the sensitivity of the results to this aspect of the prior specification, demonstrating good performance unless the departure from the independence assumption is substantial and the FVE is small.

A drawback of the current model is computational efficiency: our real-data application to the ABCD Study data took about 50 h of compute time. In future work we plan on implementing the BRAINIAC model in a more computationally efficient algorithm using a Variational Bayes algorithm. We also plan on incorporating a variety of annotations to further investigate brain–behavior relationships: while we applied the BRAINIAC model to resting state MRI associations with crystallized intelligence from the ABCD Study, the model is quite general and could be applied to a number of different modalities (e.g., task-based MRI), outcomes, and annotations (e.g., gene expression in the developing human brain). However, caution is still warranted in applying BRAINIAC to highly spatially correlated data (e.g., spatially smoothed adjacent voxels). In future work we will examine the impact of spatial smoothing on BRAINIAC estimates, e.g., using structural MRI as features.

## CRediT authorship contribution statement

**Rong W. Zablocki:** Writing – original draft, Visualization, Software, Methodology, Formal analysis. **Bohan Xu:** Writing – review & editing, Formal analysis, Data curation. **Chun-Chieh Fan:** Writing – review & editing, Formal analysis. **Wesley K. Thompson:** Writing – review & editing, Writing – original draft, Supervision, Methodology, Formal analysis, Conceptualization.

## Declaration of competing interest

The authors have nothing to declare.

## Data Availability

The authors do not have permission to share data.

## Appendix A Supplementary data

MMC S1 Conditional Posteriors and Gibbs Sampling Algorithm, Computational Resource, and MCMC coverage assessment.
