# Supplementary material for: A Bayesian Regularized and Annotation-Informed Integrative Analysis of Cognition (BRAINIAC)
Source: Dev Cogn Neurosci. 2025 Jun 25;74:101569. doi: 10.1016/j.dcn.2025.101569 (PMC12271756; doi:10.1016/j.dcn.2025.101569)
Supplement: MMC S1 — Conditional Posteriors and Gibbs Sampling Algorithm, Computational Resource, and MCMC coverage assessment. [file mmc1.pdf]

A ***B***ayesian ***R***egularized and ***A***nnotation-***In***formed ***I***ntegrative ***A***nalysis of ***C***ognition (BRAINIAC)

Rong W. Zablocki[1]
Bohan Xu[2]
Chun-Chieh Fan[2]
Wesley K. Thompson[2]

[1]Herbert Wertheim School of Public Health and Human Longevity Science, University of California San Diego, La Jolla, CA, USA
[2]Population Neuroscience and Genetics (PoNG) Center, Laureate Institute for Brain Research, Tulsa , OK, USA

1 Supplementary Materials

1.1 Conditional Posteriors and Gibbs Sampling Algorithm

Let $\hat{\boldsymbol{b}} = \frac{\boldsymbol{X}^T\boldsymbol{Y}}{n}$ denote the marginal least squares (MLS) effect size estimates and $\boldsymbol{\Lambda} = \frac{\boldsymbol{X}^T\boldsymbol{X}}{n}$ denote the in-sample correlation matrix between features.

The full conditional distributions for parameter estimates of $\boldsymbol{\beta}$, h^2 and $\boldsymbol{\alpha}$ are described below. In the posterior inference, n is the number of the subjects, N is the number of the features (equivalent to B in the main text), M is the number of annotations. The data $\boldsymbol{X}$ and $\boldsymbol{Y}$ are replaced by sufficient statistics $\hat{\boldsymbol{b}}$ and $\boldsymbol{\Lambda}$. The presentation of prior of $\boldsymbol{\beta}$ in supplementary is slightly different from that in the main text, e.g,

$$
\begin{aligned}
\boldsymbol{\beta} &\sim \mathrm{N}\left(\mathbf{0}, \frac{h^2\boldsymbol{\Psi}}{\sum_{k=1}^{N}\exp(\boldsymbol{Z}_k\boldsymbol{\alpha})}\right), \\
\boldsymbol{\Psi} &= \mathrm{diag}\{\exp(\boldsymbol{Z}_1\boldsymbol{\alpha}), \exp(\boldsymbol{Z}_2\boldsymbol{\alpha}), \ldots, \exp(\boldsymbol{Z}_N\boldsymbol{\alpha})\}.
\end{aligned}
\tag{1}
$$

Hence, $\boldsymbol{\Psi}$ is unscaled in the supplementary (so is in the Python code).

1.1.1 Posterior $\boldsymbol{\beta}$

Posterior conditional distribution of $\boldsymbol{\beta}$ has a closed form of multivariate normal distribution and can be drawn from Gibbs sampler directly:

$$
\begin{aligned}
&\boldsymbol{\beta}|\ldots \sim \mathrm{N}(\boldsymbol{\mu}, \boldsymbol{\Sigma}), \\
&\boldsymbol{\Sigma} = \frac{1-h^2}{n}\left(\boldsymbol{\Lambda} + \frac{(1-h^2)}{h^2}\frac{\sum_{k=1}^{N}\exp(\boldsymbol{Z}_k\boldsymbol{\alpha})}{n}\boldsymbol{\Psi}_{N\times N}^{-1}\right)^{-1}, \\
&\boldsymbol{\mu} = \frac{n}{1-h^2}\boldsymbol{\Sigma}\hat{\boldsymbol{b}} = \left(\boldsymbol{\Lambda} + \frac{(1-h^2)}{h^2}\frac{\sum_{k=1}^{N}\exp(\boldsymbol{Z}_k\boldsymbol{\alpha})}{n}\boldsymbol{\Psi}_{N\times N}^{-1}\right)^{-1}\hat{\boldsymbol{b}}.
\end{aligned}
\tag{2}
$$

1.1.2 Computational feasibility: block partition

Due to large dimension of $\boldsymbol{\Lambda}$ ($N \times N$), $\boldsymbol{\beta}$ can be drawn block by block in a way similar to partitioned regression. The partition of the features doesn't have to be scientifically meaningful and blocks don't have to be independent to each other. The main idea is to split large number of features into relatively

smaller blocks for computational feasibility. Let $j = 1, 2, \ldots, J$ (J is the total number of the blocks) be the index of the block, j^* be the complement blocks other than j and N_j is the total number of features within j^{th} block, then the block-wise conditional distribution of $\boldsymbol{\beta}_j$ is

$$\begin{aligned}
&\boldsymbol{\beta}_j | \ldots \sim \mathrm{N}(\boldsymbol{\mu}_j, \boldsymbol{\Sigma}_j), \\
&\boldsymbol{\Sigma}_j = \frac{1-h^2}{n} \left(\boldsymbol{\Lambda}_j + \frac{(1-h^2)}{h^2} \frac{\sum_{k=1}^{N} \exp(\boldsymbol{Z}_k \boldsymbol{\alpha})}{n} \boldsymbol{\Psi}_{N_j \times N_j}^{-1} \right)^{-1} \\
&\boldsymbol{\mu}_j = \left(\boldsymbol{\Lambda}_j + \frac{(1-h^2)}{h^2} \frac{\sum_{k=1}^{N} \exp(\boldsymbol{Z}_k \boldsymbol{\alpha})}{n} \boldsymbol{\Psi}_{N_j \times N_j}^{-1} \right)^{-1} \hat{\boldsymbol{b}^*}_j
\end{aligned} \tag{3}$$

where $\hat{\boldsymbol{b}_j^*}$ is the adjusted MLS estimate of $\hat{\boldsymbol{b}_j}$ derived as

$$\begin{aligned}
\hat{\boldsymbol{b}_j^*} &= \frac{\boldsymbol{X_j}^T (\boldsymbol{Y} - \sum_{j^* \neq j} \boldsymbol{X_{j^*}} \hat{\boldsymbol{\beta}_{j^*}})}{n} \\
&= \frac{\boldsymbol{X_j}^T \boldsymbol{Y}}{n} - \frac{\sum_{j^* \neq j} \boldsymbol{X_j}^T \boldsymbol{X_{j^*}} \hat{\boldsymbol{\beta}_{j^*}}}{n} \\
&= \hat{\boldsymbol{b}_j} - \sum_{j^* \neq j} \boldsymbol{\Lambda_{jj^*}} \hat{\boldsymbol{\beta}_{j^*}}.
\end{aligned} \tag{4}$$

The adjustment is to remove the possible effect contributed from out-j blocks. The estimated effect size $\hat{\boldsymbol{\beta}_{j^*}}$ can be obtained during MCMC iterations.

1.1.3 Posterior h^2

Posterior h^2 doesn't have a closed form and its conditional distribution is proportional (symbolized by $\propto$) to

$$\begin{aligned}
&h^2 | \ldots \propto (h^2)^{-\frac{N}{2}+a-1} (1-h^2)^{-\frac{n}{2}+d-1} \\
&\exp\left[-\frac{1}{2} \left(\frac{\{\sum_{k=1}^{N} \exp(\boldsymbol{Z}_k \boldsymbol{\alpha})\} \boldsymbol{\beta}^T \boldsymbol{\Psi}^{-1} \boldsymbol{\beta}}{h^2} + \frac{n - 2n \hat{\boldsymbol{b}}^T \boldsymbol{\beta} + n \boldsymbol{\beta}^T \boldsymbol{\Lambda} \boldsymbol{\beta}}{1-h^2} \right) \right],
\end{aligned} \tag{5}$$

h^2 is drawn via Griddy-Gibbs Sampler (GGS, [3]).

1.1.4 Posterior $\boldsymbol{\alpha}$

Posterior $\boldsymbol{\alpha}$ doesn't have a closed form, either, and its full conditional distribution is proportional to

$$\begin{aligned}\boldsymbol{\alpha}|\ldots \propto \prod_{k=1}^{N}\left\{\left[\frac{\exp(\boldsymbol{Z}_k\boldsymbol{\alpha})}{\sum_{k=1}^{N}\exp(\boldsymbol{Z}_k\boldsymbol{\alpha})}\right]^{-\frac{1}{2}}\exp\left[-\frac{\beta_k^2\sum_{k=1}^{N}\exp(\boldsymbol{Z}_k\boldsymbol{\alpha})}{2h^2\exp(\boldsymbol{Z}_k\boldsymbol{\alpha})}\right]\right\} \\ \exp\left\{-\frac{\boldsymbol{\alpha}^T\boldsymbol{\Sigma}_{\alpha}^{-1}\boldsymbol{\alpha}}{2}\right\}.\end{aligned} \tag{6}$$

Since annotations are assumed to be independent to each other, hence, $\boldsymbol{\alpha}$ can be drawn one by one via Metropolis-Hastings (MH) sampler ([1, 2]). Let $m = 1, 2, \ldots, M$, the corresponding equation (6) with respect to (w.r.t) m^{th} annotation is

$$\begin{aligned}\alpha_m|\ldots \propto \prod_{k=1}^{N}\left\{\left[\frac{\exp(Z_{k,m}\alpha_m)}{\sum_{k=1}^{N}\exp(\boldsymbol{Z}_k\boldsymbol{\alpha})}\right]^{-\frac{1}{2}}\exp\left[-\frac{\beta_k^2\sum_{k=1}^{N}\exp(\boldsymbol{Z}_k\boldsymbol{\alpha})}{2h^2\exp(\boldsymbol{Z}_k\boldsymbol{\alpha})}\right]\right\} \\ \exp\left\{-\frac{\alpha_m^2}{2\Sigma_{\alpha_{m,m}}}\right\}.\end{aligned} \tag{7}$$

t-candidate distribution is used in MH sampler with location parameter obtained by maximizing α_m over equation (7).

1.2 Computational Resource

The algorithm has been implemented in Python version 3.10 with multiprocessing invoked to run multiple MCMC chains simultaneously. Both simulations and real data application were run on Linux server with total 1 TB of RAM, dual Intel(R) Xeon(R) Gold 6342 processors, and NVIDIA(R) Tesla(R) P40 GPU accelerator (only for real data). In simulations with $\boldsymbol{X}$ dimension of $2,804 \times 14,019$, 10,000 iterations (3,000 burn-in, thinning of 10), average running time was 8.2 hours. Real data applications of ABCD with $\boldsymbol{X}$ dimension of $5,643 \times 61,776$, 10,000 iterations (3,000 burn-in, thinning of 10) took about 50 hours.

1.3 MCMC coverage assessment

Table 1: MCMC coverage of true values by setting

Settings	h^2	α_1	α_2
$\rho = 0, h^2 = 0.2$	10/10	7/10	10/10
$\rho = 0, h^2 = 0.4$	9/10	7/10	10/10
$\rho = 0, h^2 = 0.6$	9/10	7/10	10/10
$\rho = 0, h^2 = 0.8$	10/10	8/10	10/10
$\rho = 0.2, h^2 = 0.2$	8/10	7/10	8/10
$\rho = 0.2, h^2 = 0.4$	9/10	6/10	9/10
$\rho = 0.2, h^2 = 0.6$	8/10	7/10	9/10
$\rho = 0.2, h^2 = 0.8$	10/10	6/10	9/10
$\rho = 0.4, h^2 = 0.2$	6/10	6/10	10/10
$\rho = 0.4, h^2 = 0.4$	7/10	6/10	10/10
$\rho = 0.4, h^2 = 0.6$	9/10	8/10	8/10
$\rho = 0.4, h^2 = 0.8$	10/10	5/10	9/10
$\rho = 0.6, h^2 = 0.2$	1/10	8/10	7/10
$\rho = 0.6, h^2 = 0.4$	2/10	9/10	5/10
$\rho = 0.6, h^2 = 0.6$	5/10	6/10	4/10
$\rho = 0.6, h^2 = 0.8$	10/10	8/10	3/10
$\rho = 0.8, h^2 = 0.2$	0/10	5/10	5/10
$\rho = 0.8, h^2 = 0.4$	0/10	8/10	5/10
$\rho = 0.8, h^2 = 0.6$	0/10	7/10	3/10
$\rho = 0.8, h^2 = 0.8$	7/10	6/10	0/10

The denominator was the number of MCMC chains (10) per setting; the numerator was the number of chains that covered the true values of the parameters 95% of the time.

1.4 Binary Annotation (%)

Table 2: Percentage of 1's from 14 within-network binary annotations

Annotations	(%)
Auditory-Auditory	0.45
CinguloOperc-CinguloOperc	1.26
CinguloParietal-CinguloParietal	0.02
Default-Default	1.33
DorsalAttn-DorsalAttn	0.80
FrontoParietal-FrontoParietal	0.45
None-None	1.75
RetrosplenialTemporal-RetrosplenialTemporal	0.05
SMhand-SMhand	1.14
SMmouth-SMmouth	0.05
Salience-Salience	0.01
Subcortical-Subcortical	0.28
VentralAttn-VentralAttn	0.41
Visual-Visual	1.20

The denominator of the percentage is $B = 61,776$.

References

[1] W Keith Hastings. Monte carlo sampling methods using markov chains and their applications. *Biometrika*, 57:97–109, 1970.

[2] Nicholas Metropolis and Stanislaw Ulam. The monte carlo method. *Journal of the American statistical association*, 44(247):335–341, 1949.

[3] Christian Ritter and Martin A Tanner. Facilitating the gibbs sampler: the gibbs stopper and the griddy-gibbs sampler. *Journal of the American Statistical Association*, 87(419):861–868, 1992.
